# *AtEXP2* Is Involved in Seed Germination and Abiotic Stress Response in *Arabidopsis*

**DOI:** 10.1371/journal.pone.0085208

**Published:** 2014-01-03

**Authors:** An Yan, Minjie Wu, Limei Yan, Rui Hu, Imran Ali, Yinbo Gan

**Affiliations:** 1 Zhejiang Key Lab of Crop Germplasm, Department of Agronomy, College of Agriculture and Biotechnology, Zhejiang University, Hangzhou, Zhejiang, China; 2 Institute of Hydrobiology, Chinese Academy of Sciences, Wuhan, Hubei, China; University of Michigan, United States of America

## Abstract

Expansins are cell wall proteins that promote cell wall loosening by inducing pH-dependent cell wall extension and stress relaxation. Expansins are required in a series of physiological developmental processes in higher plants such as seed germination. Here we identified an *Arabidopsis* expansin gene *AtEXPA2* that is exclusively expressed in germinating seeds and the mutant shows delayed germination, suggesting that *AtEXP2* is involved in controlling seed germination. Exogenous GA application increased the expression level of *AtEXP2* during seed germination, while ABA application had no effect on *AtEXP2* expression. Furthermore, the analysis of *DELLA* mutants show that *RGL1*, *RGL2*, *RGA*, *GAI* are all involved in repressing *AtEXP2* expression, and *RGL1* plays the most dominant role in controlling *AtEXP2* expression. In stress response, *exp2* mutant shows higher sensitivity than wild type in seed germination, while overexpression lines of *AtEXP2* are less sensitive to salt stress and osmotic stress, exhibiting enhanced tolerance to stress treatment. Collectively, our results suggest that *AtEXP2* is involved in the GA-mediated seed germination and confers salt stress and osmotic stress tolerance in *Arabidopsis*.

## Introduction

The dominant phase of the life cycle of higher plants initiates from seed germination. The embryo of the *Arabidopsis* seed is surrounded by a single-cell endosperm layer and testa (seed coat) [1]–[2], thus, the emerging radicle needs to overcome the dual constraint of these structures to complete germination, therefore seed germination is defined as a two stage process with testa rupture followed by endosperm rupture [1], [3]. Physiologically the rupture of endosperm and testa is associated with cell division and enlargement, which are accompanied by cell wall expansion and loosening [4]–[5]. The cell wall is an extracellular layer surrounding the cell that plays an important role in maintaining cell shape and provides mechanical strength and rigidity [4]. The cell wall is composed of cellulose microfibrils, hemicellulose, pectin, lignin, and proteins, these cell wall polymers interact together to form a polymeric network then to confer the structural rigidity to cell wall [4]. Therefore, cell wall enlargement needs to destroy this structural rigidity by modifying cell wall extensibility.

Many enzymes are involved in modifying matrix polysaccharides including endo-β-mannanase and other endoglucanases [6]. In addition to those enzymes, expansins are involved in cell wall extensibility modification [7]–[8]. Expansin was firstly identified from young cucumber seedlings for its ability to mediate the acid-induced extension of cucumber hypocotyl walls [7], [9]. Expansins induce cell wall extension by disrupting non-covalent linkages between cellulose microfibrils and the cross-linking matrix glycans in cell wall [8]–[10].

Expansins are encoded by a multigene family and highly conserved in gymnosperms and angiosperms [10]–[12]. Expansins can be divided into four divergent subfamilies based on genomic and phylogenetic analyses, denoted as α-expansin (EXPA), β-expansin (EXPB), EXP-like A (EXLA) and EXP-like B (EXLB) [10], [13], and there are thirty-six expansins encoding by 26 *EXPA*s, 6 *EXPB*s, 3 *EXLA*s and 1 *EXLB*s in *Arabidopsis* (http://www.personal.psu.edu/fsl/ExpCentral).

The extensive occurrence of the expansins superfamily in plants suggests that expansins play multiple roles during the plant life cycle [14]–[15], and in the past several decades numerous studies have accumulated evidence about their involvement in diverse developmental processes, including seed germination [16]–[17], seedling morphogenesis [18], root architecture [19]–[26], leaf development [27]–[30], fruit ripening [31]–[32], stoma opening [33]–[34], pollination [35], abscission, stress responses [36]–[43] and others. However, those characterized expansins only represent a small proportion of the expansins superfamily in the plant kingdom, and the individual functions of other expansin coding genes still remains to be elucidated.

In the present study, we investigated the expression pattern of *AtEXP2* in *Arabidopsis*, found that the expression of *AtEXP2* is associated with seed germination and subject to GA and stress regulation. Our molecular analyses demonstrate that *AtEXP2* plays a key role in controlling seed germination through GA signaling.

## Materials and Methods

### Plant Materials and Growth Conditions

*Arabidopsis thaliana* ecotype Columbia-0 and Landsberg erecta were used as the wild types in this study. The T-DNA insertion mutant *exp2* (Salk_117075) was obtained from the Nottingham *Arabidopsis* Stock Centre (NASC). The *ga1-3*, *rgl1-1*, *rgl2-1*, *rga-t2*, *gai-t6* and *della* multiple mutants have been described previously [44]–[46]. The plants were grown in a growth room under a 16-h-light (100 µmol·m^−2^·s^−1^, 22°C) and 8-h-dark (19°C) photoperiod.

### Germination Assay and Stress Treatment

For germination assays, *Arabidopsis* seeds were surface-sterilized with 5% (v/v) NaClO solution for 10 min and washed five times with sterile water, then sown on 1/2 MS medium containing 0.8% (w/v) agar supplemented with or without additional sucrose (150 or 250 mM), NaCl (100 or 200 mM), mannitol (200 or 400 mM), paclobutrazol (1 or 5 µM), then plates were transferred to a tissue culture room at 22°C under a 16 h-light/8 h-dark photoperiod. Germination rates were scored daily until the 7th day after sown based on radicle tip emergence. Experiments were repeated three times on five plates with about 100 seeds for each genotype.

### GA and ABA Treatments

For measuring the effect of GA applications on gene expression, Col-0 wild type seeds were imbibed in 10 µM GA_3_ (Sigma-Aldrich), then were harvested 4 h, 6 h and 8 h after treatment for RNA extraction respectively. For measuring the effect of ABA applications on gene expression, Col-0 seeds were imbibed in different concentrations of ABA (0.1, 0.3, 1, 3, 10 µM) (Sigma-Aldrich) and harvested 16 h after treatment for RNA extraction.

### Construction of Transgenic Lines

All the constructs (*35S:AtEXP2*, *pAtEXP2:GUS*) were prepared using the Gateway technology (Invitrogen). For construct *35S: AtEXP2*, the *EXP2* coding region was amplified from cDNA using primers 5′-CGGTCGACTACTCATCCCCTTTTCCAC-3′ and 5′-AAGCGGCCGCCTAAAATTGTCCGCCTTC-3′. The PCR products were digested with Sal I and Not I and cloned into pENTR-1A vector (Invitrogen), then recombined into destination vector pK2GW7 using the Gateway LR reaction (Invitrogen). To construct *pEXP2:GUS*, the 1.8 kb genomic fragment upstream of the start codon in *EXP2* was amplified with the primers 5′-CGGTCGACAAGAAGTATCTGGGTGGG-3′ and 5′-AAGCGGCCGCATGGGCTAAAGAGGAGGA-3′, the digested PCR product was cloned into pENTR-1A and recombined into pHGWFS7. All binary vector constructs were introduced into *Agrobacterium* strain GV3101 and transformed into *Arabidopsis* Columbia-0 using the floral dip method [47]. All the transgenic lines were first selected based on their antibiotics resistance and further confirmed by expression level of the *EXP2* or histochemical GUS assays.

### RNA Extraction and Real-time RT-PCR

Total RNAs were isolated from *Arabidopsis* seeds according to the protocol described previously [48], and total RNAs from other tissues were extracted using TRIZOL reagent (Invitrogen) following the manufacturer’s instructions. cDNA was synthesized using the M-MLV Reverse Transcriptase (Promega) from 2 µg of total RNA in a 25 µl reaction, and diluted 4-fold with water. Quantitative real-time RT-PCR was performed using SYBR-green as described in previous study [49]–[51]. *TUB2* was used as an endogenous control gene to normalize expression of the other genes. Primers used in real-time RT-PCR as follows : *EXP2*-F: 5′-CATAAACTCCGACGACAACG-3′, *EXP2*-R: 5′-TACCCACAAGCACCACCCAT-3′; *TUB2*-F: 5′-ATCCGTGAAGAGTACCCAGAT-3′, *TUB2*-R: 5′-AAGAACCATGCACTCATCAGC-3′ [52]. The PCR program was as follows: 30 s at 95°C, followed by 40 cycles of 5 s at 95°C, 30 s at 60°C.

### GUS Activity Analysis

Tissues were prefixed in 90% acetone on ice for 20 min and incubated in GUS staining buffer [50 mM sodium phosphate (pH 7.0), 10 mM EDTA, 1 mg/ml 5-bromo-4-chloro-3-indoyl-β-D-glucuronide, 0.5 mM potassium ferricyanide, and 0.5 mM potassium ferrocyanide, 0.1%(v/v) Triton X-100] at 37°C overnight. Stained samples were then cleared of chlorophyll in an ethanol series and photographed by light microscopy.

## Results

### *AtEXP2* is Mainly Expressed in Germinating Seeds

**Figure 1:**
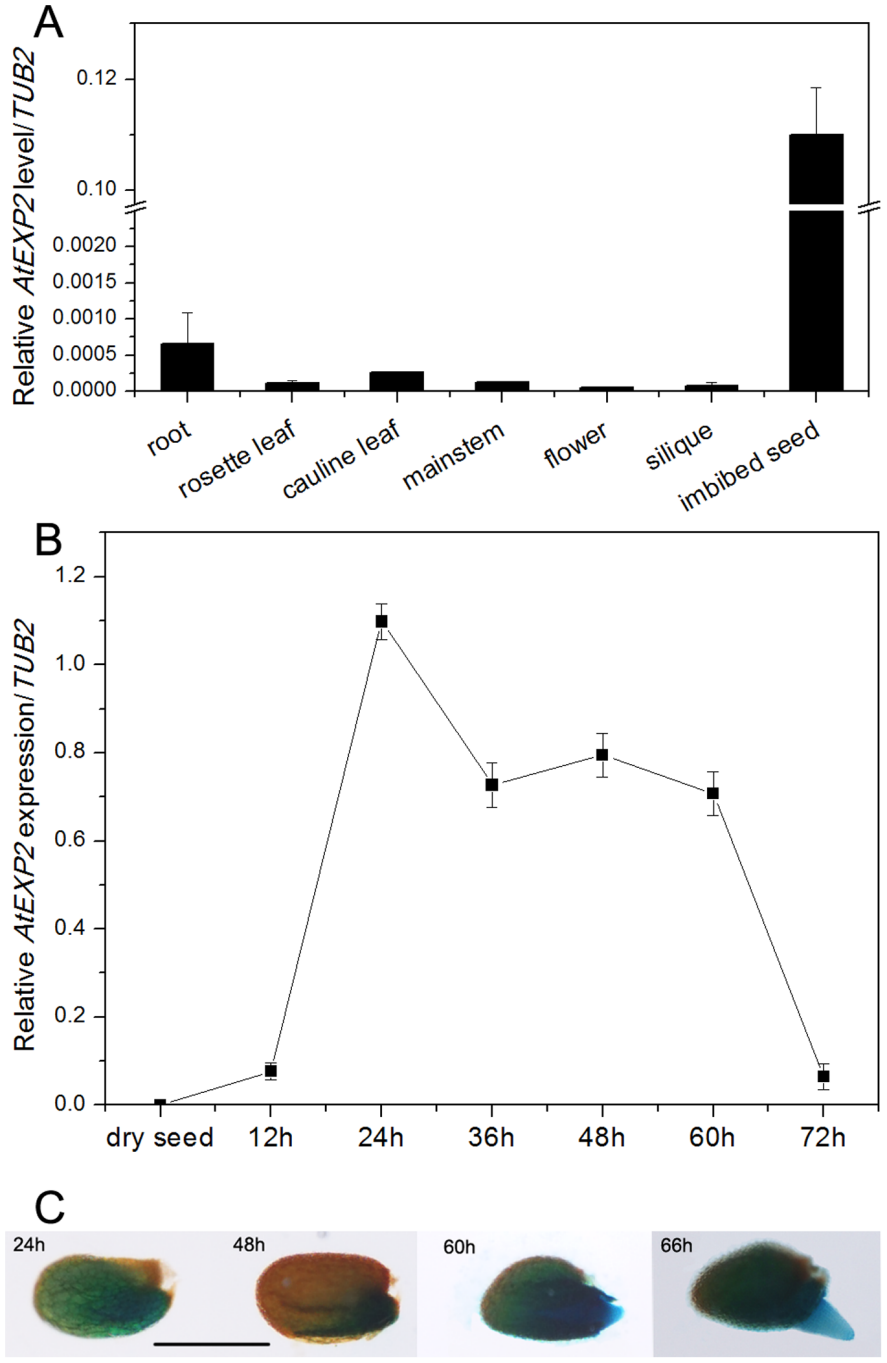
Expression pattern of *AtEXP2* gene. (A) Tissue-specific expression of *AtEXP2*. Various tissues of *Arabidopsis* wild type plants were harvested for RNA extraction. (B) Time course of *AtEXP2* expression. Dry seeds and imbibed seeds of Col-0 were harvested for RNA extraction. Transcript levels of *AtEXP2* were measured by real-time RT-PCR, and the values were normalized against the levels of *TUB2* as a control. Error bars represent SD. (C) GUS staining in germinating seeds of the *pAtEXP2:GUS* transgenic line. Seeds from T_3_ homozygous plants of the *pAtEXP2:GUS* transgenic line were analyzed. Bar = 1 mm.

By analyzing the available public *Arabidopsis* microarray database (http://www.bar.utoronto.ca/efp/cgi-bin/efpWeb.cgi), we discovered that only *AtEXP2* was exclusively expressed in imbibed seeds among thirty-six *Arabidopsis* expansin coding genes, which implies a probable role of *AtEXP2* in seed germination. Therefore we first examined the expression pattern of *AtEXP2* in *Arabidopsis* by real-time RT-PCR, and we found a high expression level in germinating seeds, but very low level in other tissues including roots, rosette leaves, cauline leaves, mainstem, flowers and siliques (Figure 1A). Further analysis showed that the expression of *AtEXP2* was not detected in dry seeds, but the transcript abundance in seeds began to accumulate after imbibition in water, and the expression level reached a peak after 24 h imbibition, and then remained at high levels 2–3 days after imbibition (Figure 1B). These observations suggest that *AtEXP2* is a seed-specific gene and may be involved in the seed germination process.

Next, we cloned the 1.8 kb genomic sequence upstream of transcription start site of *AtEXP2* and generated the *pAtEXP2:GUS* construct. The β-glucuronidase (GUS) assays in this transgenic line shows *AtEXP2* promoter activity continuously detectable during seed imbibition in water (Figure 1C). Later, GUS staining was observed in the radicle. These results are consistent with the RT-PCR assays and further suggests that *AtEXP2* plays a role in seed germination and possibly root function.

### *AtEXP2* is Required in Seed Germination

**Figure 2:**
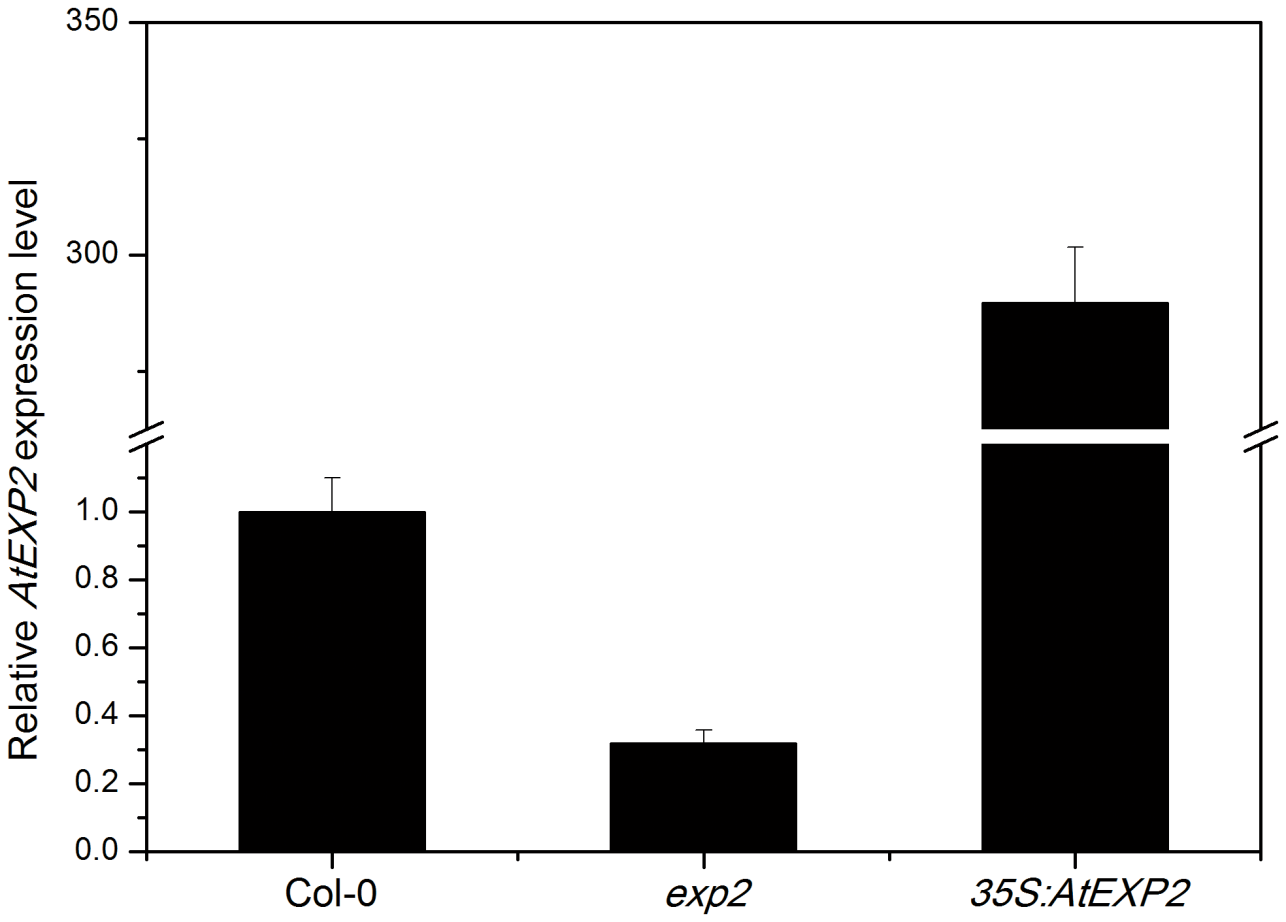
Relative expression levels of *AtEXP2* in *exp2* and *35S:AtEXP2* line. Seeds of wild type, *exp2* and *35S:AtEXP2* overexpression line were harvested for RNA extraction after 24 h imbibition in water. Transcript abundance was measured by real-time RT-PCR and the values were normalized against the levels of *TUB2* as a housekeeping gene. Error bars represent SD.

**Figure 3:**
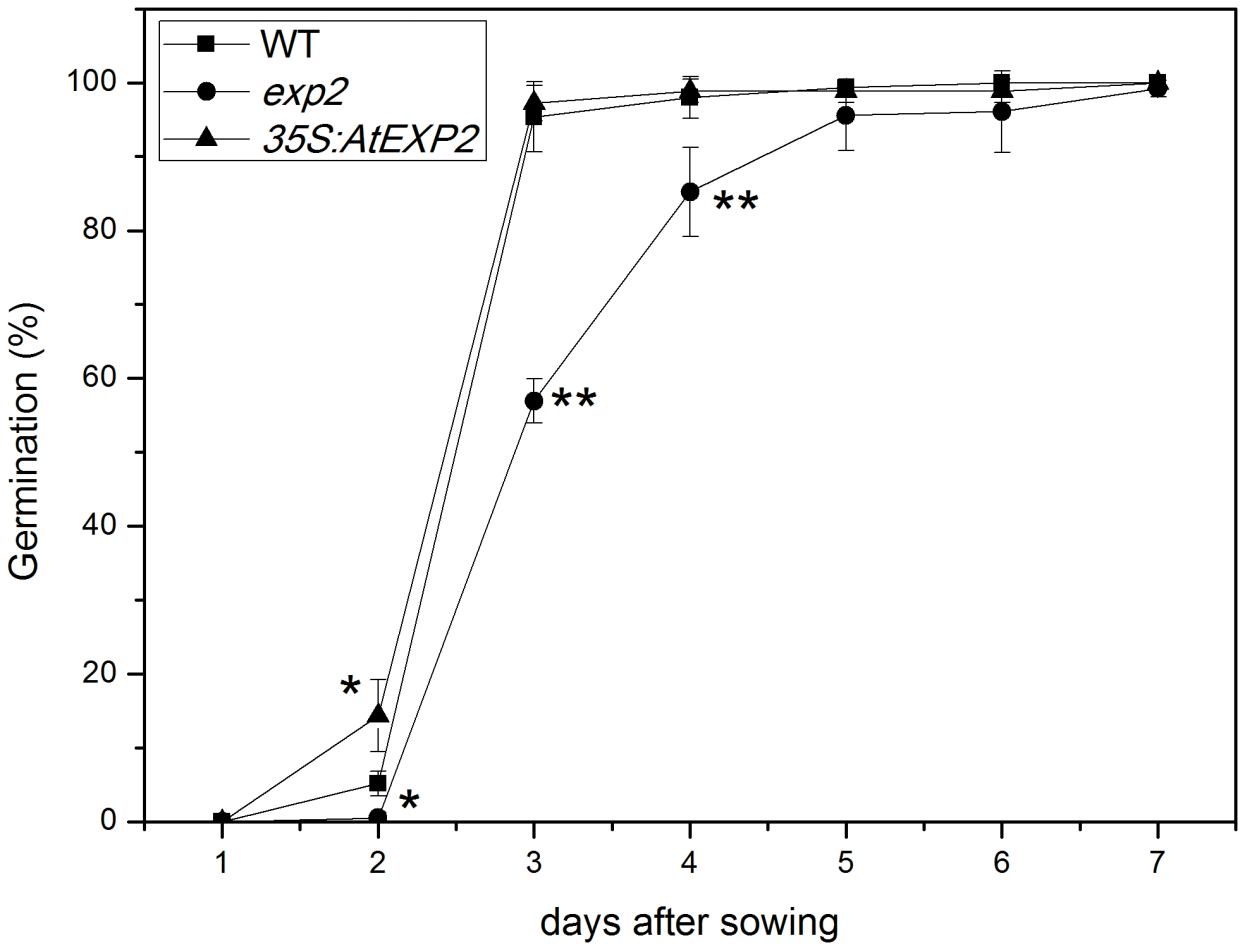
Germination phenotype of the wild type, *exp2* and *35S:AtEXP2* line. Non-dormant seeds of wild type, *exp2* and *35S:AtEXP2* overexpression line were employed in the germination assay. The germination frequencies were scored daily until the 7th day after sown. Error bars represent SD. A Student’s t-test was calculated at the probability of either 5% (*P<0.05) or 1% (**P<0.01).

In order to investigate whether *AtEXP2* plays a role in seed germination, we isolated an *exp2* mutant (Salk_117075) carrying a T-DNA insertion in the *AtEXP2* promoter region, which almost completely suppresses *AtEXP2* expression (Figure 2). We also created *35S:AtEXP2* overexpression transgenic lines and generated nine independent transgenic lines, from which we selected a representative transgenic line exhibiting significantly higher *AtEXP2* expression level compared to wild type (Figure 2). Then we performed the germination assay using seeds of homozygous *exp2* mutant and *35S:AtEXP2* line, the results showed that *35S:AtEXP2* line germinated much earlier than wild type on the second day after sown on plates, while the rate of germination in *exp2* mutant was significantly delayed compared to wild type (Figure 3), suggesting that *AtEXP2* is required for seed germination in *Arabidopsis*.

### The Participation of *AtEXP2* in Seed Germination is Regulated by GA

**Figure 4:**
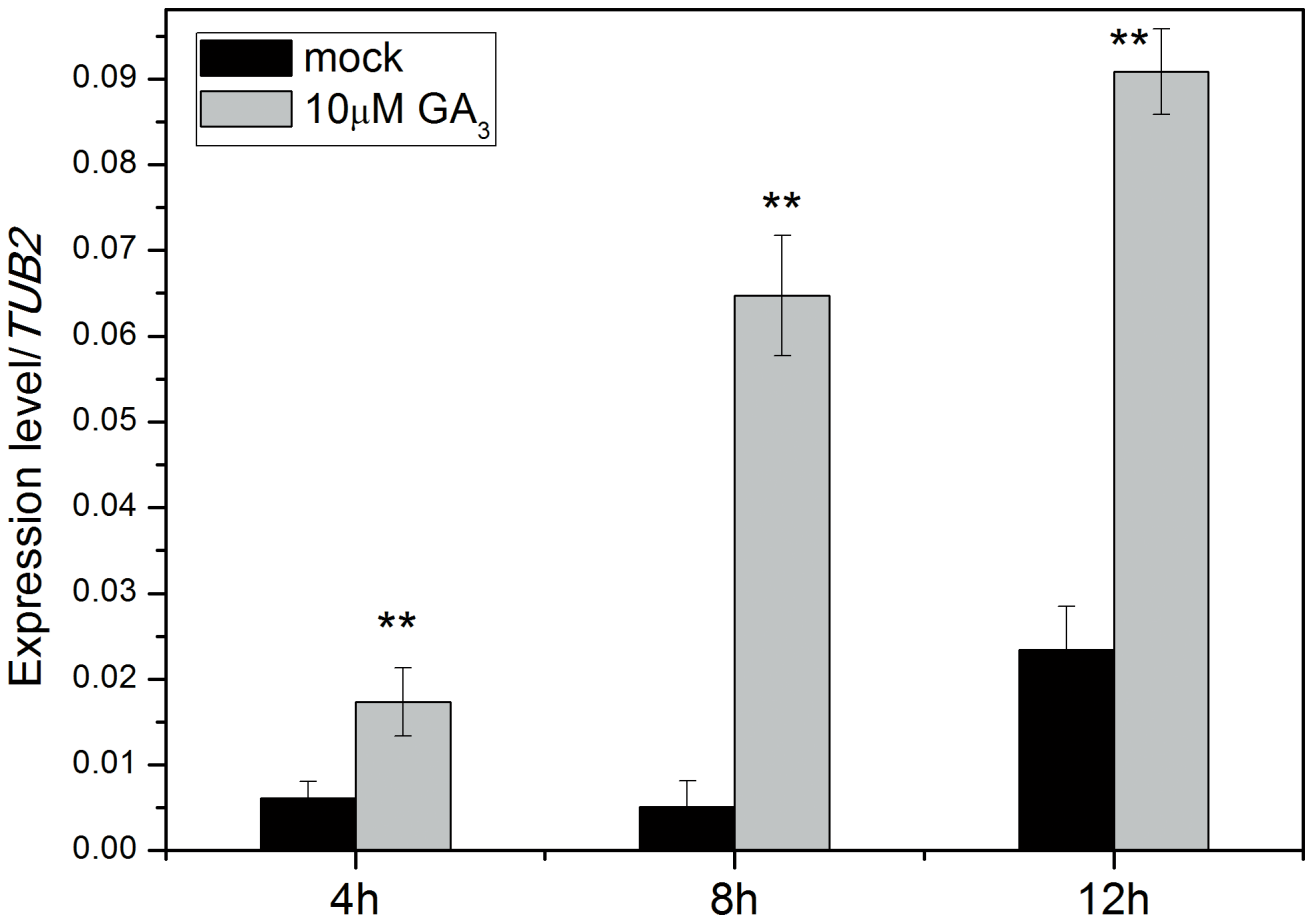
Expression of *AtEXP2* in response to exogenous GA application. Col-0 wild type seeds were harvested 4 h, 6 h and 8 h after imbibition in 10 µM GA_3_ solution for RNA extraction respectively. Transcript levels were measured by real-time RT-PCR, and the values were normalized against the levels of *TUB2* as a control. Error bars represent SD. A Student’s t-test was calculated at the probability of 1% (**P<0.01).

**Figure 5:**
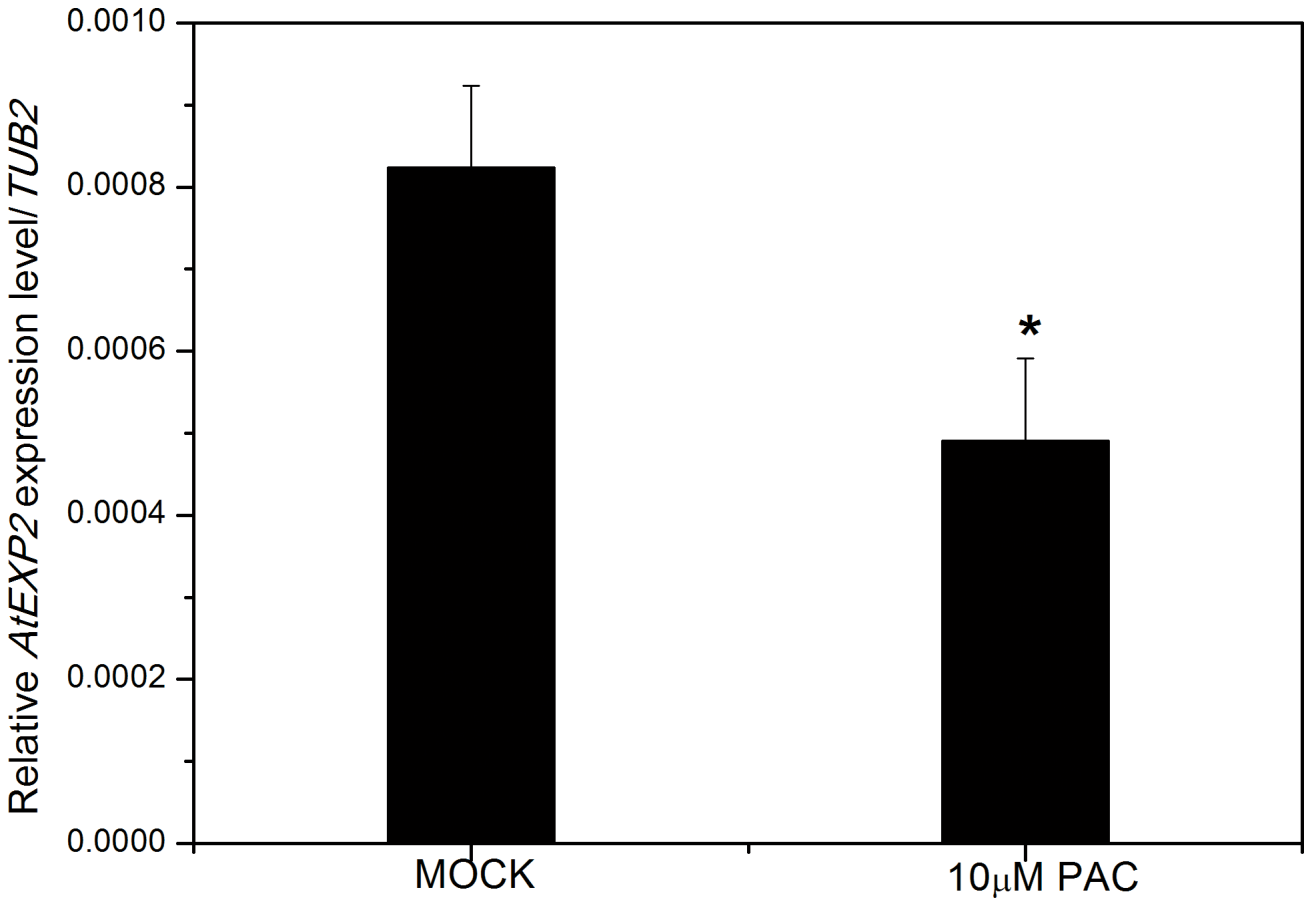
Expression of *AtEXP2* in response to paclobutrazol treatment. *AtEXP2* expression was determined by quantitative real-time RT-PCR in 24 h imbibed seeds treated with 10 µM PAC or without (Mock). Error bars represent SD. A Student’s t-test was calculated at the probability of 5% (*P<0.05).

Since seed germination is largely controlled by phytohormones GA and ABA [2], [53], we next determined whether the expression of *AtEXP2* was regulated by GA and ABA. As shown in Figure 4, the expression of *AtEXP2* was significantly higher in GA_3_-treated wild type seeds than in mock-treated seeds. After treating seeds with 10 µM paclobutrazol (PAC), a gibberellin biosynthesis inhibitor, the expression of *AtEXP2* was significantly decreased compared to the mock treatment (Figure 5), suggesting that the expression of *AtEXP2* is induced by GA in germinating seeds. In contrast to GA induction of *AtEXP2* expression, the expression of *AtEXP2* was not significantly affected when the seeds were treated with different concentration of ABA (data was not shown).

**Figure 6:**
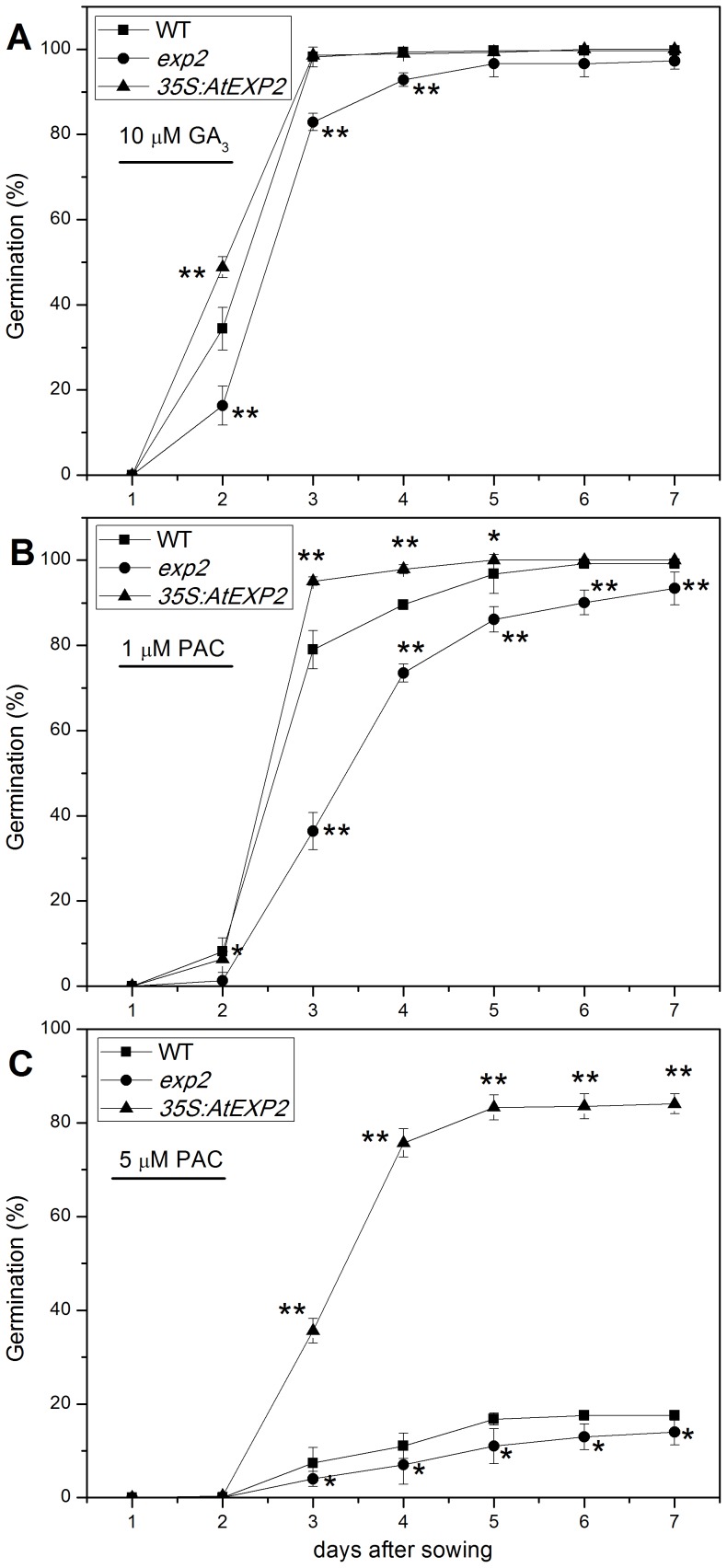
Germination phenotype of the wild type, *exp2* and *35S:AtEXP2* line in response to GA and PAC treatment. Seeds of wild type, *exp2* and *35S:AtEXP2* line were treated with 10 µM GA_3_ (A), 1 µM (B) or 5 µM (C) paclobutrazol (PAC). Error bars represent SD. A Student’s t-test was calculated at the probability of either 5% (*P<0.05) or 1% (**P<0.01).

In order to investigate whether *AtEXP2* is involved in seed germination in response to GA, we examined the germination phenotype of wild type, *exp2* mutant and overexpression line in the present of GA_3_ and paclobutrazol, as shown in Figure 6A, the germination rate of *exp2* seeds was significantly lower than that of wild type seeds on the second day after sown on plates when exogenous GA was applied, while the *35S:AtEXP2* line exhibited significant higher germination rate than wild type, indicating that *AtEXP2* likely controls seed germination through GA signaling. However, in the presence of 1 µM and 5 µM paclobutrazol, the *exp2* seeds still showed significant lower germination rate than wild type, while the germination of *35S:AtEXP2* line was less inhibited by paclobutrazol compared to wild type (Figure 6B and 6C), implied that factors other than GA would be involved in the *AtEXP2*-mediated seed germination.

**Figure 7:**
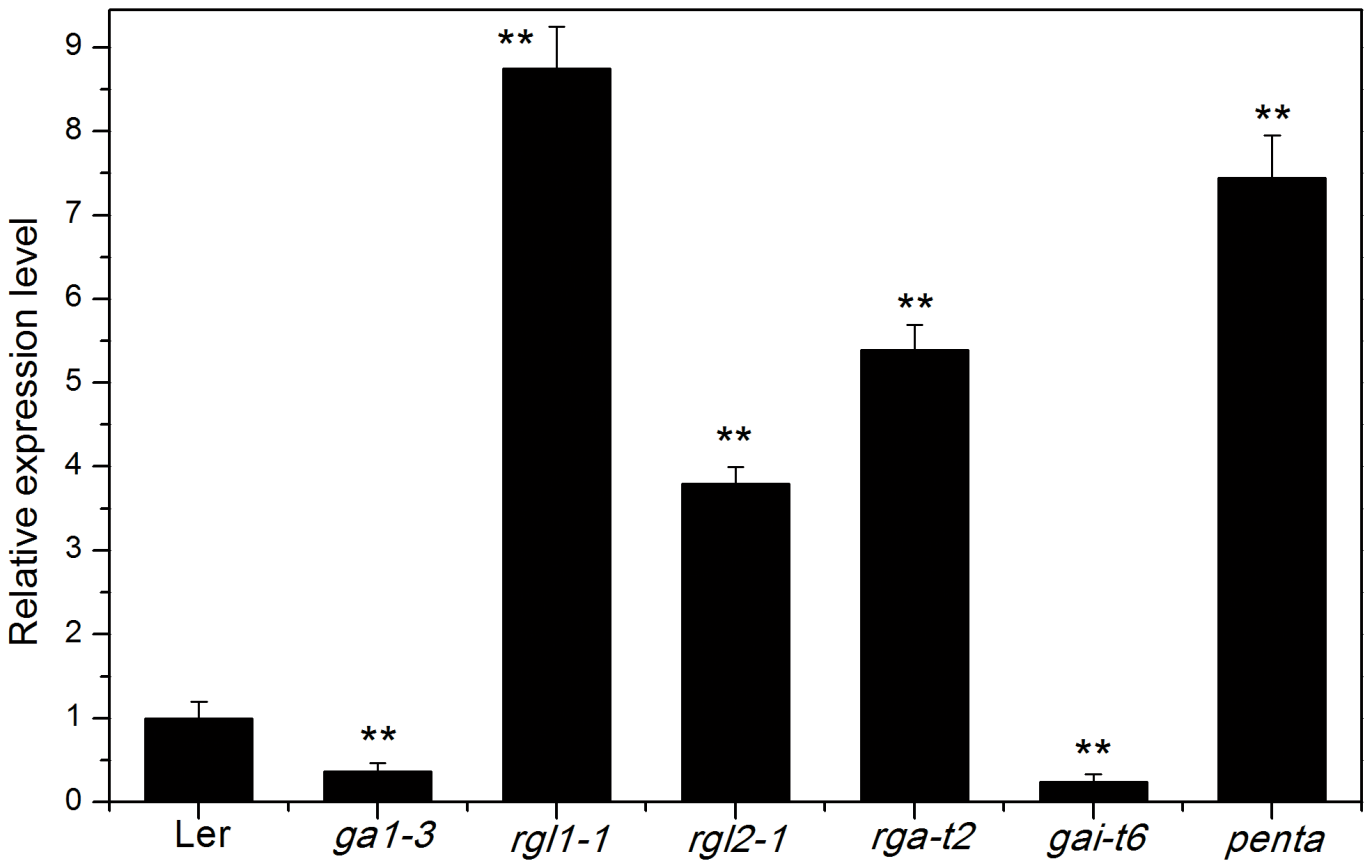
Effects of DELLA on *AtEXP2* expression during seed germination. Expression level of *AtEXP2* was measured in 24 h imbibed seeds of wild type, *ga1-3*, and various *DELLA* mutants. *penta* indicates the *ga1-3 gai-t6 rga-t2 rgl1-1 rgl2-1* mutant. Error bars represent SD. A Student’s t-test was calculated at the probability of 1% (**P<0.01).

To further elucidate the way *AtEXP2* participates in GA signaling during seed germination, we next examined the expression level of *AtEXP2* in the GA-deficient mutant *ga1-3* and various *DELLA* mutants. As shown in Figure 7, *AtEXP2* expression was significantly reduced in *ga1-3*, confirming our previous conclusion that the *AtEXP2* expression was induced by GA. *AtEXP2* expression was also reduced in semi-dominant gain-of-function *DELLA* mutant *gai-t6* seeds compared to wild type, while other three *DELLA* mutants *rgl1-1*, *rgl2-1* and *rga-t2* all exhibited significant higher expression of *AtEXP2* than wild type, with *rgl1-1* shown the highest expression level. These results suggest that all four *DELLA* genes tested in the study contribute to the repression of *AtEXP2* expression and *RGL1* plays the principal role in controlling *AtEXP2* expression. However in the *penta* mutant, which lacks all four DELLA proteins activities in *ga1-3* background, the expression of *AtEXP2* was lower than that in *rgl1-1* (Figure 7), indicating that other regulators besides these four DELLA proteins would be involved in the GA-mediated *AtEXP2* expression.

### *AtEXP2* is Involved in Response to Salt Stress and Osmotic Stress in Seed Germination

**Figure 8:**
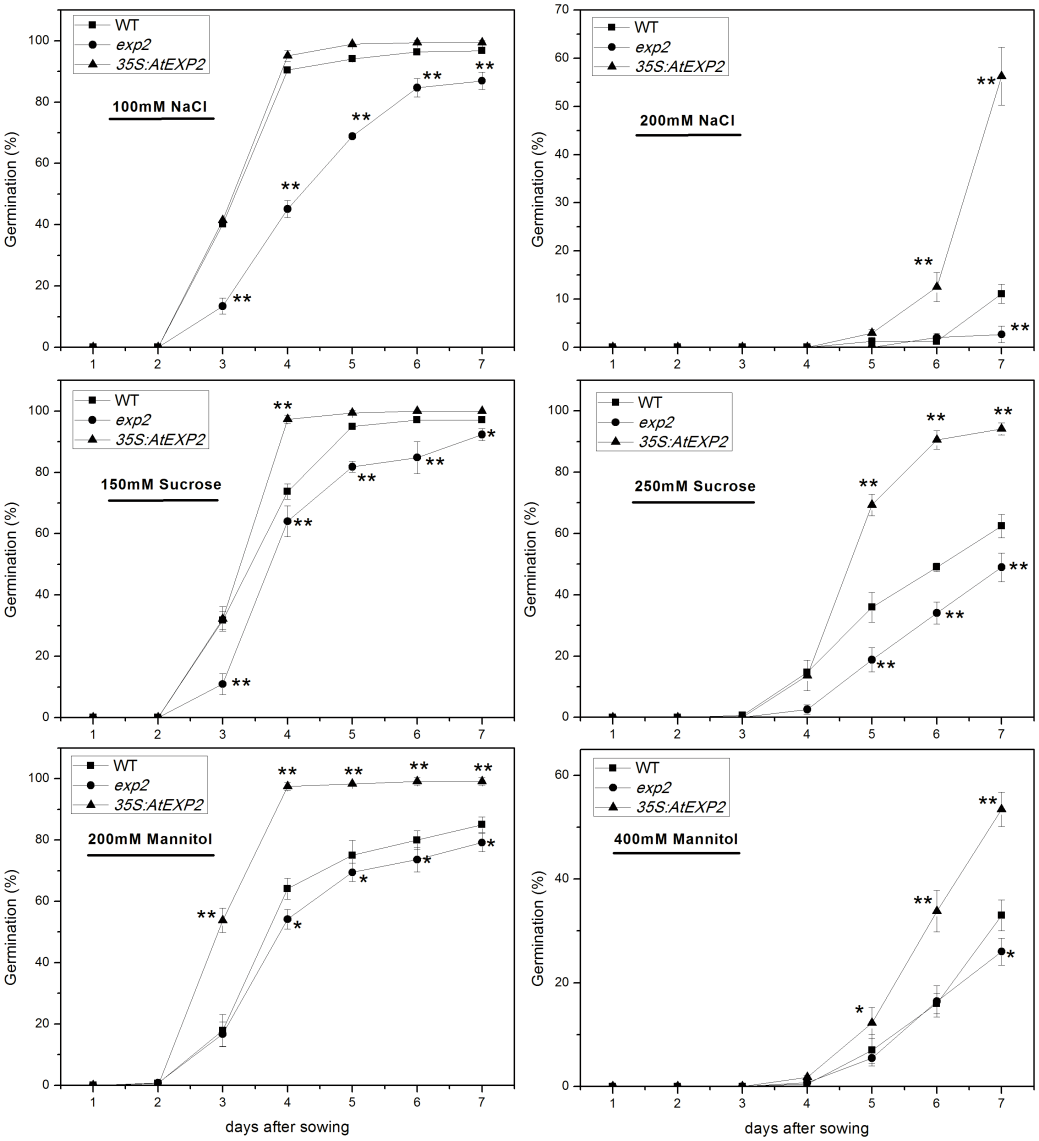
Germination phenotype of the wild type, *exp2*, and *35S:AtEXP2* line in response to abiotic stresses. Seeds of wild type, *exp2* and *35S:AtEXP2* line were treated with different concentrations of NaCl (100 or 200 mM), sucrose (150 or 250 mM) and mannitol (200 or 400 mM). Error bars represent SD. A Student’s t-test was calculated at the probability of either 5% (*P<0.05) or 1% (**P<0.01).

The expression of expansin coding genes are not only regulated by developmental signals, but also affected by environmental cues [17], [54]. In order to investigate whether *AtEXP2* is involved in response to abiotic stress, we performed germination tests under salt stress and osmotic stress condition. As shown in Figure 8, when exposed to 100 mM NaCl, the germination frequency of *exp2* mutant seeds was much lower than wild type and overexpression line, and ultimately reached a proportion less than 90% at seven days after sown. In the high salt condition (200 mM), germination of all genotypes was severely affected, with less than 15% germination frequency in wild type, and less than 5% germination frequency in *exp2*. Interestingly, the germination frequency of *35S:AtEXP2* line still reached approximately 60% seven days after sowing, suggesting that *AtEXP2* was involved in seed germination in response to salt stress.

Next, we considered the possible effect of osmotic stress on *AtEXP2* expression. Including various concentrations of sucrose and mannitol to the MS plates, we observed significantly lower germination frequency in the *exp2* mutant compared to the wild type. The overexpression line was much higher in these conditions, moreover, the differences were more evident when under higher concentrations of sucrose and mannitol (Figure 8). Together, these observations indicate that *exp2* mutant is more sensitive to salt stress and osmotic stress than wild type, while the *35S:AtEXP2* overexpression line is less sensitive than wild type, showing elevated tolerance to salt stress and osmotic stress.

**Figure 9:**
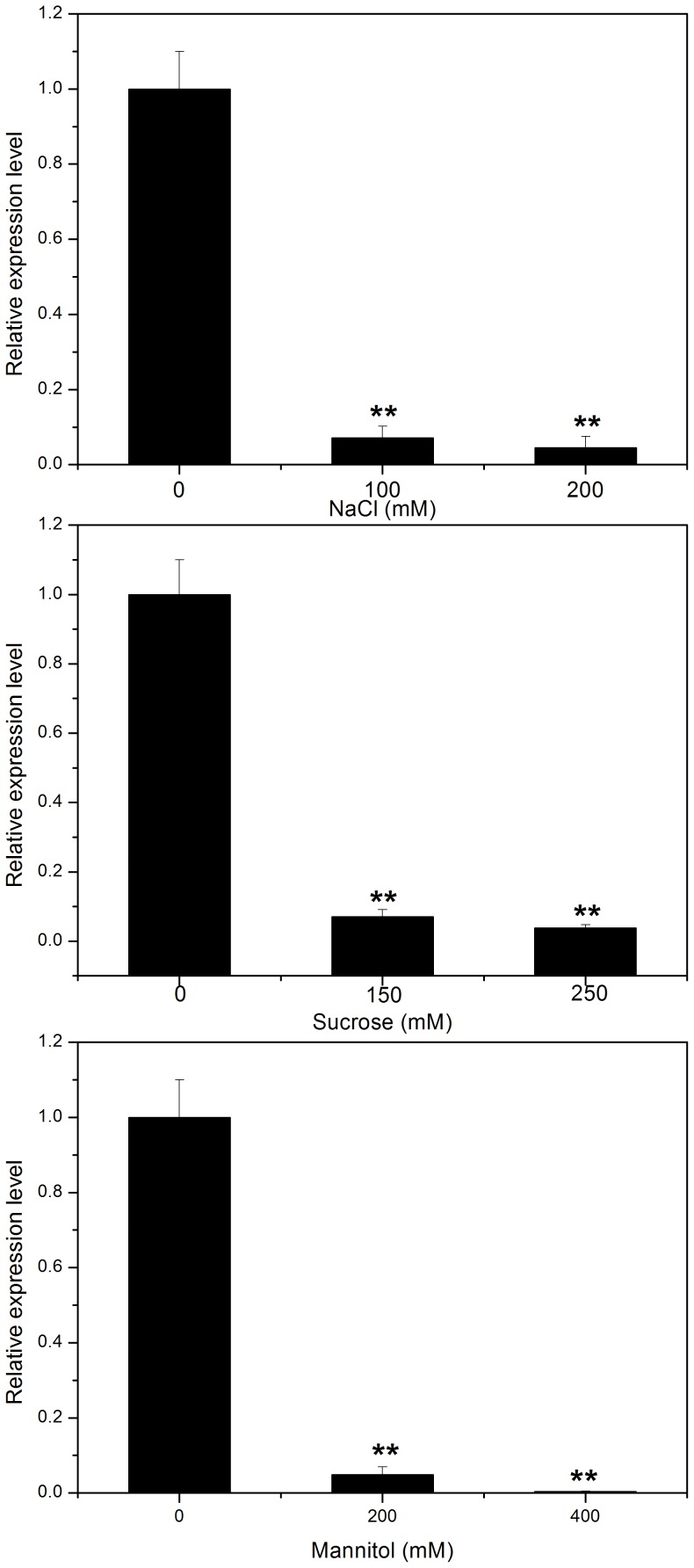
Expression of *AtEXP2* in response to salt and osmotic stresses during seed germination. Col-0 seeds were collected 24 h after imbibition in different concentrations of NaCl, sucrose and mannitol. Error bars represent SD. A Student’s t-test was calculated at the probability of 1% (**P<0.01).

In order to further study the function of *AtEXP2* in response to abiotic stress in seed germination, we performed quantitative real-time PCR analysis to evaluate *AtEXP2* expression in response to salt stress and osmotic stress in germinating wild type seeds, the results showed that the expression of *AtEXP2* were remarkably reduced after NaCl, sucrose and mannitol treatments compared to control treatments, and the reduction of expression levels were more severe when the seeds were treated with increased concentrations of NaCl, sucrose and mannitol (Figure 9), suggested that abiotic stress repressed the *AtEXP2* expression in germinating seeds. In summary, *AtEXP2* overexpression could confer salt and osmotic stress tolerance in *Arabidopsis* seed germination, and the salt stress and osmotic stress inhibit seed germination by repressing the expression of *AtEXP2*.

## Discussion

Expansins are encoded by a large gene superfamily and are widely distributed in plant species, consistent with the possibility that expansins perform multiple functions in various aspects of plant life cycle among species. Previous studies have shown that expansins were involved physiologically in almost every developmental process during the plant life cycle, related to cell growth, cell separation and cell wall disassembly [4], [55]–[56], and different expansins would play diverse tissue-specific roles distinct from each other [54]–[55], [57]–[58]. Physiological change during seed germination is associated with cell enlargement and cell wall expansion, and previous studies in tomato have revealed a role in seed germination played by expansins [16]–[17]. Furthermore, *LeEXP4* is expressed specifically in the micropylar endosperm cap region and associated with endosperm cap weakening [17]. Another two expansin genes *LeEXP8* mRNA is localized to the radicle cortex of the embryo, and *LeEXP10* mRNA is expressed throughout the embryo during seed germination, suggesting the specific roles of the two expansin genes during seed germination [16]. In the present study, we first used the public *Arabidopsis* microarray database to analyze the expression profile of all thirty-six expansin genes in *Arabidopsis* and found that only *AtEXP2* was exclusively expressed in germinating seeds, further real-time RT-PCR analyses confirmed that *AtEXP2* was specifically expressed in imbibed seeds and *AtEXP2* mRNA amounts peaked after 24 hours in imbibed seeds and maintained a high level during early stage of seed germination, consistent with previous microarray data [59]–[62], Using a β-glucuronidase reporter fusion to the *AtEXP2* promoter, we observed signals in the germinating seeds, consistent with the *AtEXP2* RNA accumulation pattern.

In order to investigate the role of *AtEXP2* during seed germination, we carried out a germination assay using *exp2* mutant seeds, which showed that *exp2* seeds germinate later than wild type. Further through analyzing promoter sequence of *AtEXP2* using PLACE tools (Plant cis-acting regulatory DNA elements) [63] we found there were putative GA-responsive elements (GARE) and ABA-responsive elements (ABRE) in the promoter region of *AtEXP2*, indicated that *AtEXP2* would function downstream of GA and ABA signaling. We then performed GA and ABA treatments and found *AtEXP2* expression was GA-inducible, but not affected by ABA treatment, which was consistent with previous studies [1], [17], [60], [64]. GA is well known to be a pivotal phytohormone in breaking seed dormancy and promoting seed germination [1]–[2], [53]. Previous studies showed that active GAs are synthesized mainly in the radicle and micropylar endosperm during germination [60], stimulating growth potential of embryo and inducing hydrolases biosynthesis to weaken endosperm and other structures surrounding the embryo, thus allow the radicle emergence and complete seed germination [65]–[66]. In this context, we suggest that *AtEXP2* is involved in GA-mediated promotion of seed germination by weakening cell wall in endosperm and related structures surrounding the embryo.

As the GA signaling pathway is mainly mediated by derepression of DELLA repressors [67]–[68], Stamm et al., (2012) have shown that RGL2 downregulates two expansin encoding genes *EXPA3* and *EXPA8* in seed germination [69]. In order to study whether *AtEXP2* expression is subjected to the regulation of DELLA, we examined the *AtEXP2* expression level in germinating seeds of various *DELLA* mutants and found that all four DELLA genes tested participated in the repression of *AtEXP2* expression, including *RGL1*, *RGL2*, *RGA* and *GAI*. Moreover, *RGL1* played the most dominant role in controlling *AtEXP2* expression. Taken together, our results suggest that GA promote *AtEXP2* expression by removing the repression effect of DELLA on *AtEXP2* during seed germination.

In addition to the regulation by phytohormones, expansin genes are also differentially regulated by various environmental cues [54], such as abiotic stresses including salt stress, heat stress, drought stress, and water stress [30], [37], [43], [70]. Here we tested the germination phenotype of *exp2* and the overexpression line in response to salt stress and osmotic stress, and we found that *exp2* mutant exhibited hypersensitivity to salt stress and osmotic stress compared to wild type, while the overexpression line showed reduced sensitivity to these stress treatments and revealed enhanced tolerance to stress to a certain extent. These results suggest that *AtEXP2* may confer salt and osmotic stress tolerance in *Arabidopsis* seed germination. Real-time RT-PCR analysis showed that salt stress and osmotic stress treatments significantly reduced the expression level of *AtEXP2*, implying that salt stress and osmotic stress may inhibit seed germination by repressing *AtEXP2*-mediated cell wall expansion. In contrast to our observation, some other expansin coding genes in other species are subject to upregulation by stress [37], [43], [71]. This variable gene-specific expression pattern of expansin genes under abiotic stress suggest that individual expansin genes are subjected to different regulation by stress conditions and that the mechanisms underlying them are distinct.
